# Characterization of the complete chloroplast genome of *Dalbergia cultrata* (Leguminosae)

**DOI:** 10.1080/23802359.2019.1631131

**Published:** 2019-07-12

**Authors:** Yu Liu, Ping Huang, Chang-Hong Li, Feng-Qi Zang, Yong-Qi Zheng

**Affiliations:** State Key Laboratory of Tree Genetics and Breeding, Laboratory of Forest Silviculture and Tree Cultivation, Research Institute of Forestry, Chinese Academy of Forestry, Beijing, China

**Keywords:** *Dalbergia cultrata*, chloroplast genome, phylogenetic analysis

## Abstract

*Dalbergia cultrata* is a Near Threatened species with high ecological and economic values. In this study, its chloroplast genome was assembled using Illumina pair-end sequencing dataset. The chloroplast genome has a quadripartite structure with 156,385 bp in length and contains a pair of 16,392 bp inverted repeat (IR) regions, which were separated by large single copy (LSC: 86,040 bp) region and small single copy (SSC: 37,561 bp) region. A total of 121 genes were annotated, including 77 protein-coding genes (PCGs), 36 tRNAs, and 8 rRNAs. The overall GC content was 36.1%. The phylogenetic analysis revealed that *D. cultrata* has close relationship to *D. hainanensis* and *D. odorifera*. This complete chloroplast genome can be readily used for population genetic studies of *D. cultrata*.

*Dalbergia cultrata* is a deciduous arbor species belonging to the genus *Dalbergia* of family Faboideae, with high ecological and economic values due to its resistances to disease, insect and fire, and precious hardwood timber. This species was distributed in a tropical and subtropical zone in Indo-China peninsula, and the south of Yunnan province in China. It is well known and valued for the quality and beauty of its heartwood (Wang et al. 2016). However, this species was threatened by overexploitation and land use change. Information of chloroplast genomes has been extensively applied to understanding plant genetic diversity and conservation genetics, while studies regarding this plant are lacking (Li et al. 2019). In this study, we assembled the complete chloroplast genome of *D. cultrata* based on the whole-genome Illumina sequencing dataset.

Seeds of *D. cultrata* were collected from Simao County, Yunnan Province, China (100°41′E;22°57′N) and sowed in experimental greenhouse of Chinese Academy of Forestry; after then, fresh leaves were collected from 1-year-old seedlings. The voucher specimen was deposited in Research Institute of Forestry, Chinese Academy of Forestry in Beijing, China.Genomic DNA was isolated with improved CTAB protocol (Doyle and Doyle 1987) and sequencing using the Illumina HiSeq X Ten (Majorbio, Shanghai, China). In total, 21.4 million high quality base pairs of sequence data were obtained and used for the chloroplast genome assembly using SOAPdenovo v2.04. The resulting contigs were linked based on overlapping regions after being aligned to *D. odorifera* (MF_668133) (Wariss et al. 2018). Annotation was performed using Blastp, coupled with manual adjustment. Finally, the circular genome map was generated with Organellar Genome DRAW. The annotated chloroplast genome of *D. cultrata* has been deposited into the GenBank with the accession number MK599253.

The complete chloroplast genome of *D. cultrata* was 156,385 bp in length. It has a typical quadripartite structure, including a large single copy (LSC) region of 86,040 bp and a small single copy (SSC) region of 37,561 bp with a pair of inverted repeat (IR) regions of 16,392 bp. The overall GC content was 36.1%. It annotated 121 genes, including 77 protein-coding genes (PCGs), 36 tRNA genes, and 8 rRNA genes. Among these, seven genes contain a single intron while two genes possess two introns. Seven protein-coding genes, six tRNAs genes, and four rRNAs genes were duplicated in both IR regions.

To explore the evolution status of *D. cultrata*, as well as Faboideae, we inferred the phylogenetic relationships based on the complete chloroplast genomes of fifteen species. The phylogenetic tree was constructed with RaxML based on fourteen complete chloroplast genome sequences of Faboideae and *Albizia odoratissima* as outgroup (Figure 1). All sequences were aligned using MAFFT (Nakamura et al. 2018) and using maximum-likelihood (ML) analysis based on the Tamura–Nei model in MEGA v 10.0.5. The phylogenetic analysis revealed that *D. cultrata* has close relationship to *D. hainanensis* and *D. odorifera*. This complete chloroplast genome can be readily used for population genetic studies of *D. cultrata*.

**Figure 1.**
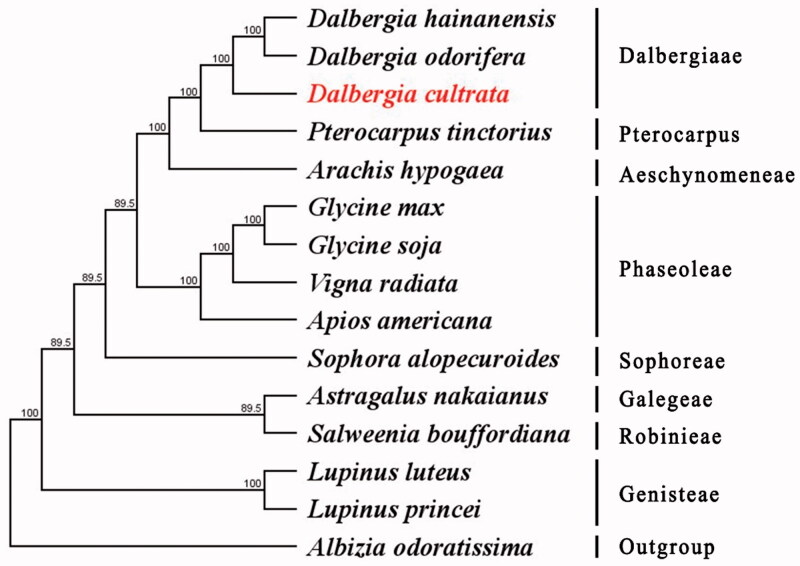
Maximum-likelihood (ML) tree based on the complete chloroplast genome sequences of *D. cultrata* and other 14 species. Numbers in the nodes are the bootstrap values from 1000 replicates. Their accession numbers are as follows: *Albizia odoratissima*: NC_034987; *Apios americana*: NC_025909; *Arachis hypogaea*: NC_037358; *Astragalus nakaianus*: NC_028171; *Dalbergia cultrata*: MK599253; *Dalbergia hainanensis*: NC_036961; *Dalbergia odorifera* isolate Y801: MF668133; *Glycine max*: NC_007942; *Glycine soja*: NC_022868; *Lupinus luteus*: NC_023090; *Lupinus princei*: KU726829; *Pterocarpus tinctorius*: MH033829; *Salweenia bouffordiana*: MF449303; *Sophora alopecuroides*: NC_036102; *Vigna radiata*: NC_013843.
